# Observations on the Remuneration of Medical Practitioners

**Published:** 1812-06

**Authors:** 


### *To the Editors of the Medical and Physical Journal.*

GENTLEMEN,

IN the whole range of desiderata which crowd upon the mind of every liberal man who has engaged himself in the general practice of medicine, there is not one subject which so forcibly calls upon his attention, or by which he is so frequently led to deplore the present imperfect state of his profession, as the mode by which he and his brother labourers labourers are rewarded for the sacrifices of time, and the exertions of skill, which they daily make in the service of their fellow-creatures. It is not that this sensation is excited by any selfish feeling of interest, although the surgeon and apothecary in the country is indeed paid very inadequately, but it is impossible that he should not, every day, lament the necessity of adopting a line of conduct, which alone can enable him to reap those advantages which his education, his talents, and his necessities, imperiously demand. He sees the physician, little employed indeed, but remunerated liberally in proportion to the attendance which he bestows upon his patients; whilst he is frequently obliged either to consume his time in unproductive visits, or to do violence to his own honesty, and must often run the risk of offending his patient, by ordering for him medicines in an expensive form, which, to say the least of them, might very well be dispensed with. But it is unnecessary to enlarge upon this topic; it is notorious that the whole country is full of complaints, that they cannot send for their medical attendant on any trivial occasion, without being drenched, as they call it, with nauseous drugs. We must agree to the truth of this assertion, but not to the justice of the galling insinuations with which it is urged. It is difficult to make them think in opposition to their own interests; but let any one observe the labour which it costs an apothecary, even in the best lines of country practice, to make a return in his ledger of 1000l. a year, and then, when he has deducted about 30 per cent. for expenses, let him say from his heart whether he has been overpaid. He will not then, perhaps, urge that at the beginning of a dangerous complaint, when double attendance is often required, that the practitioner charges him four or five shillings every day for what may not cost one-fourth of that sum. He will remember that, after the first danger is over, there are weeks of convalescence, where almost the same quantity of time is occupied, but where little or perhaps no medicine is taken; he will remember also, that in the surgical department the most troublesome and tedious dressings are daily performed, for months, without any medicine being ordered, and therefore where the charge for attendance is still less adequate than in the most unproductive cases of the former kind; and, finally, he may recollect how often days and nights of anxiety are passed in cases of midwifery, for which, and the month's visiting, a few shillings only are received.

In this state of mind, I would ask him how much he thinks, upon the average, a medical man receives for every visit which he pays in the course of a day. I feel almost convinced, convinced, he would say, that such things stood in need of reform, and would even offer to reward his surgeon as he does his physician, in the exact ratio of his attendance; and thus establish between them a system which would stimulate industry to exertion on the one part, and on the other, establish a confidence that the medicines taken were necessary for the cure of the complaint for which they were ordered.

To the medical man, such a reform has very long appeared of the greatest consequence; but the imaginary evils of *reform* universally appear so gigantic in the dim gloom of uncertainty, that to make a beginning becomes considerably more than half the battle; and hence, I am sure, that too many expressions of thanks cannot be offered to those medical men of Kendal, who have circulated the resolutions to which they have agreed, on this subject, in your Journal for January last. This is a spark which ought to communicate its influence so extensively, that the whole medical population shall feel its effects, and I doubt not that you will receive, ere long, multiplied accounts of similar plans being adopted throughout the kingdom. I shall only trespass further on your time by saying, that, should the change become general, all ranks of people will have reason to feel grateful for the spirited exertions of the surgeons of Kendal.

I am, Gentlemen,

Your's, &c. H.

P.S. I have foreborne to make any remarks upon the Kendal scheme, although it is very imperfect; but it would be a most essential benefit if some of your able correspondents would organize some extensive arrangement which might be adopted by the whole country.

